# Bioactive Metabolites of *Dioscorea* Species and Their Potential Applications in Functional Food Development

**DOI:** 10.3390/foods14142537

**Published:** 2025-07-20

**Authors:** Pengcheng Wang, Yashi Wang, Shiqi Liu, Kai Wang, Yuxuan Yao, Weizhen Liu, Donghui Li, Wei Wang, Bin Li, Yupei Yang

**Affiliations:** TCM and Ethnomedicine Innovation & Development International Laboratory, School of Pharmacy, Hunan University of Chinese Medicine, Changsha 410208, China; pengchengw3@126.com (P.W.); 18173724730@163.com (Y.W.); shiqiliu670@163.com (S.L.); wangkai161@163.com (K.W.); 18573685303@163.com (Y.Y.); q2724034830@163.com (W.L.); 15674946991@163.com (D.L.); wangwei402@hotmail.com (W.W.)

**Keywords:** *Dioscorea*, functional foods, bioactive metabolites, nutraceuticals, disease prevention

## Abstract

*Dioscorea* species, known as “Yams”, belong to the *Dioscoreaceae* family. Members of the *Dioscoreaceae* family are widely distributed across subtropical and tropical regions. They are notable for their high content of starch, dietary fiber, and various bioactive compounds. In addition to serving as a staple food source, these tubers possess significant medicinal value in traditional medicine, particularly for treating diabetes, diarrhea, and various inflammatory diseases. This study aimed to comprehensively summarize the active components and food development potential of Dioscorea species from research over the past decade by searching commonly used databases such as PubMed, Web of Science, Scopus, and Google Scholar. This review highlights the classification of bioactive compounds in *Dioscorea* spp. using the NPClassifier tool. We discuss 60 representative bioactive metabolites, including terpenoids, phenylpropanoids, carbohydrates, fatty acids, alkaloids, and amino acids. Additionally, we discuss the functional food applications and regulations of *Dioscorea* spp., which possess antioxidant, anti-inflammatory, anti-diabetic, and anticancer properties. This review is expected to provide scientific ideas for future research related to prioritizing the optimization of extraction technologies, the execution of rigorous clinical trials to confirm therapeutic effects, and the exploration of novel applications of *Dioscorea* spp. bioactives to fully harness their potential in improving human health.

## 1. Introduction

The functional food sector is undergoing a gradual transformation within the food industry, characterized by the replacement of synthetic additives with bioactive compounds derived from natural plant sources [1]. Representative examples of such plants include *Abelmoschus esculentus* [2], beetroot [3], *Polygonatum* spp. [4], medicinal mushrooms [5,6], citrus fruits [7], and *Dioscorea* spp. [8].

Yam tubers (*Dioscorea* spp.) are a significant staple in many regions due to their high content of starch, dietary fiber, and non-starch soluble sugars, and their low lipid concentration. In China, they serve as an important source of caloric energy, while in Africa and South America, they constitute a staple food for millions, thereby playing a significant role in enhancing food security [9]. Post-harvest, yam tubers should be stored in cool, shaded, and dry environments, with the ambient relative humidity maintained at 90–95% and temperatures between 29–32 °C [10]. *Dioscorea* spp. are rich in bioactive components, such as polysaccharides [11], diosgenin [12], allantoin [13], alkaloids [14], and polyphenols [15], which have been associated with various health-promoting and disease-preventive properties [16]. This review seeks to offer an extensive summary of the bioactive components of *Dioscorea* spp. and to evaluate their functional roles in the food system. The objective is to support the rational development and application of *Dioscorea* in the formulation of functional food products.

## 2. Methodology

Information on the *Dioscorea* spp. was retrieved using scientific search engines, including PubMed, Web of Science, Scopus, and Google Scholar. Using keywords such as “*Dioscorea* spp.”, “functional foods”, “*Dioscorea* species”, “*Dioscorea* foods”, “nutritional and therapeutic value of *Dioscorea*”, “clinical trials and *Dioscorea*”, “bioactive components”, and “health benefits of *Dioscorea*”, we collected and reviewed relevant studies and data published over the past decade. All chemical structures were drawn using ChemDraw 22.0.

This review systematically summarizes the bioactive components identified in natural products derived from *Dioscorea*. Through frequency analysis and rigorous data screening, important metabolites with high recurrence across studies were identified.

Traditional approaches to classifying the bioactive components of *Dioscorea* have largely relied on manual review and expert judgment. However, these methods present inherent limitations, including a lack of standardization, inconsistent classification results, and limited capacity to elucidate structure–activity relationships. To overcome these shortcomings, this review employs a deep learning-based method for structural classification of compounds using the NPClassifier tool. This tool employs deep neural network modeling trained on extensive structural datasets, enabling the automated recognition and hierarchical classification of complex chemical structures. Compounds are classified according to pathway, super class, and class, with metabolic pathways classified into seven major categories: fatty acids, polyketides, shikimates-phenylpropanoids, terpenoids, alkaloids, amino acids/peptides, and carbohydrates [17]. By employing NPClassifier, this study aims to establish a more standardized, accurate, and scalable framework for the structural classification of bioactive metabolites in species of *Dioscorea*.

## 3. Bioactive Metabolites of *Dioscorea* spp.

*Dioscorea* spp. are rich in a variety of metabolites, most of which exhibit notable biological activity [18]. Table 1 presents a detailed list of 60 representative compounds, compiled through a comprehensive literature analysis, frequency analysis, and systematic screening from recent research on *Dioscorea* spp.

### 3.1. Terpenoid Compounds

The *Dioscorea* spp. comprise the following primary terpenoids: diterpenes, triterpenes, and specific steroids. Experimental in vitro results exhibited that this compound contains anti-inflammatory, anti-diabetic, and uric acid-lowering functional activities [31]. Our analysis further revealed that *Dioscorea* spp. compounds containing spirostane steroid skeleton, such as dioscin (Compound No. 2, Table 1) and gracillin (Compound No. 5, Table 1), show promise for roles in functional food supplements, with therapeutic capacity to treat metabolic disorders and prevent cancer pathogenesis, through their ability to significantly regulate glycolipid metabolism and confer anti-tumor effects. In our previous studies, we established that the natural products of spirostane steroids, particularly diosgenin (Compound No. 1, Table 1), have been extensively investigated.

**Figure 1:**
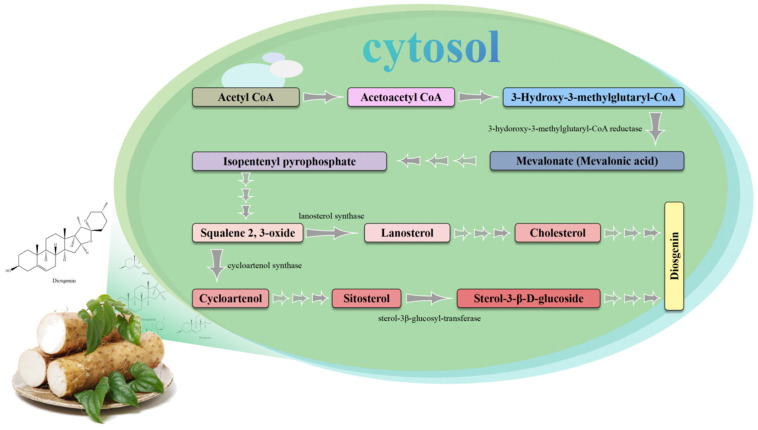
Schematic of diosgenin biosynthetic pathway.

The NPClassifier tool categorizes diosgenin as a spirostane steroid. Notably, spirostane steroids exhibit hydroxyl substitution at site 3β, a double bond at sites 5–6, and an R-configuration at position 25. Diosgenin exhibits inherent therapeutic ability, including antimicrobial, anticancer, and antioxidant [23]. Recent research on diosgenin has established that it has a significant ameliorative effect on dysmenorrhea and premenstrual syndrome in women [21]. Notably, diosgenin obtained from the genus *Dioscorea* is typically used in the initial process during the commercial synthesis of various steroids, including cortisone, pregnenolone, and progesterone [110,111]. The biosynthesis of diosgenin in *Dioscorea* spp. is a highly complex metabolic pathway involving multiple enzyme-catalyzed reactions. In the initial phase, acetyl-coenzyme A (acetyl-CoA) is converted into isopentenyl diphosphate (IPP) and dimethylpropenyl diphosphate (DMAPP), which are the precursors of diosgenin elements. This conversion occurs either through the mevalonate (MVA) or the methylerythritol phosphate (MEP) pathways. Following the synthesis of farnesyl pyrophosphate from IPP and DMAPP catalyzed by farnesyl diphosphate synthase (FPS), FPP is subsequently converted into 2,3-oxido2,3-oxidosqualene by the sequential action of squalene epoxidase (SE) and squalene synthase (SS). The resulting 2,3-oxysqualene serves as a key precursor for sterol biosynthesis, undergoing cyclization to lanosterol via lanosterol synthase (LSS), or to cycloartenol via cycloartenol synthase (CAS), depending on the biosynthetic route. Subsequently, cyclopineol is converted into cholesterol via enzymatic reactions involving several steps [22]. Figure 1 presents the various processes involved in the diosgenin biosynthesis pathway. Existing research indicates that a potential candidate gene (CYP94D144) belonging to the CYP450 gene family may be a significant regulator of the concentration levels of diosgenin in *Dioscorea* spp. [20]. An in-depth exploration of the roles of these genes and the corresponding enzymes holds the potential to provide a robust theoretical basis for large-scale synthesis and clinical utility of diosgenin.

### 3.2. Shikimate and Phenylpropanoid Compounds

The primary metabolites of shikimates and phenylpropanoids compounds in *Dioscorea* spp. comprise flavonoids, phenanthrenes, phenolic acids (C6–C1), diarylheptanoids, stilbenes, phenylpropanoids (C6–C3), and coumarins. These substances offer significant therapeutic potential [112].

**Figure 2:**
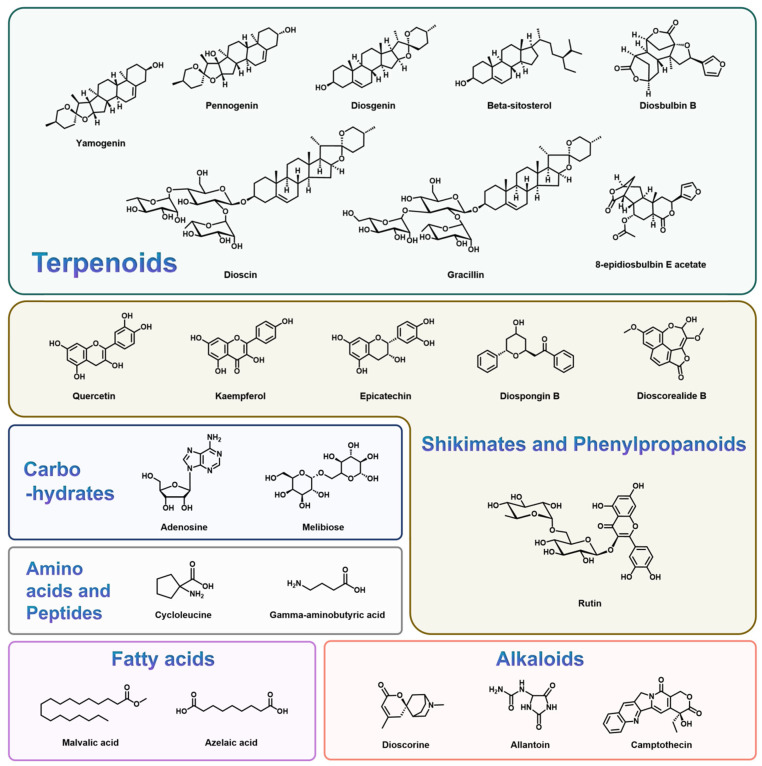
The structures of the main bioactive compounds in *Dioscorea*.

Notably, the primary structural composition of flavonoids comprises a series of compounds consisting of two phenolic hydroxyl benzene rings (A- and B- B-rings) interlinked through the three centrally positioned carbon atoms. These compounds are interlinked with phenolic hydroxyl, methoxy, methyl, isopentenyl, and other functional groups, such as quercetin (Compound No. 15, Table 1). Figure 2 presents the structure of flavanoids. Flavanoids are vital secondary metabolites in *Dioscorea* spp., with critical roles in various bioactivities including anti-tumor, anti-inflammatory, immunomodulatory, neuroprotective, hypoglycemic, and hypolipidemic functions [113,114,115].

Phenanthrene compounds identified in *Dioscorea* spp. have been demonstrated to exhibit significant antioxidant properties [116,117], which vary across various parts of the plant, with pulp and pericarp shown to exhibit superior antioxidant abilities [118,119]. Additionally, the phenanthrene compounds identified in the genus *Dioscorea* exhibit significant potential in functional ability, including dual inhibition of α-glucosidase and protein tyrosine phosphatase 1B, which play a significant role in the management of diabetes mellitus, alongside other metabolic syndromes [107,120]. Notably, phenanthrene compounds obtained from the yam skin show high cyclooxygenase (COX) enzyme inhibitory activity compared to non-steroidal anti-inflammatory drugs (NSAIDs) [121,122]. Additionally, they exhibit anticancer properties and non-cytotoxicity to cervical [123] and lung cancer cells [117].

Structurally, phenolic acid (C6-C1) compounds are defined by the presence of a carboxyl group and one or more hydroxyl groups attached to the benzene ring. Notably, the genus *Dioscorea* is relatively rich in phenolic acids, including chlorogenic acid, butyric acid, vanillic acid, p-hydroxybenzoic acid, and p-coumaric acid.

Diarylheptanoids typically comprise compounds with two aromatic rings linked via a seven-carbon chain. Importantly, diarylheptanoids are vital bioactive components used in traditional medicine to treat various diseases, with their applications in pharmaceuticals, the food industry, and cosmetology gaining significant attention [124]. Diarylheptanoic acid analogs identified in *Dioscorea* spp. can participate in the metabolic regulation of adipocytes, thereby improving glucose utilization, while inhibiting lipidogenesis, thereby providing a framework on how to mitigate the global obesity health burden [125,126]. Additionally, diarylheptanoic acid compounds may serve as vital candidates involved in anti-pancreatitis, as well as in the prevention of pancreatic necrosis [127].

Stilbenoids exhibit a similar structural identity to phenanthrenes; in particular, both contain a 1,2-diphenylethene backbone structure, with varying substituents within their composition structure. Dihydroresveratrol (Compound No. 41, Table 1) is a compound obtained from *Dioscorea dumetorum* [77].

Phenylpropanoids, characterized by one or more C6-C3 structural units in their core skeleton, are a class of secondary metabolites with diverse biological functions. In *Dioscorea* spp., research on phenylpropanoid compounds remains limited. Existing related studies have mainly focused on rosmarinic acid (Compound No. 38, Table 1), which has been identified in relatively high concentrations in *Dioscorea* spp. leaves and is believed to contribute significantly to their antioxidant activity [69].

### 3.3. Carbohydrate Compounds

In diabetic dietary therapy, yam starch has shown a low glycemic index, indicating its potential to provide a more gradual postprandial blood glucose response and therefore offering a viable nutritional option for individuals with diabetes [86]. Additionally, yams contain non-starch polysaccharides—one of their primary bioactive compounds—which play the role of antioxidants, thickeners, and stabilizers within the food industry [128,129]. Modern phytochemical and pharmacological analyses have established that yam polysaccharides have a variety of pharmacological effects, contributing to their widespread utilization. Research has shown that polysaccharides obtained from *yam bulbils* (PYBs) can effectively improve energy metabolism and reduce oxidative stress by up-regulating hepatic glycogen content and elevating antioxidant enzyme activities, such as superoxide dismutase and glutathione peroxidase. These effects lead to potent antioxidant and anti-fatigue activities [44]. Additionally, research findings have shown that carbohydrates can interact with other bioactive compounds to exert pharmacological effects. Yam glycoprotein, a component extracted from Chinese yam (*Dioscorea opposita*) and comprising primarily sugars and proteins, has exhibited significant anti-inflammatory and immunomodulatory effects [43,130].

### 3.4. Fatty Acid Compounds

The lipid compounds in *Dioscorea* spp. primarily include free fatty acids, conjugates, and fatty acid esters. Fatty acids are carboxylic acids comprising a long hydrocarbon chain and a carboxyl group. The hydrocarbon chain is often a linear structure with a length ranging from a few to multiple tens of carbon atoms. Fatty acid esters are compounds generated via the esterification process of fatty acids with alcohols. In a study involving the extraction of methanol extract from *Dioscorea bulbifera* leaves obtained from Endau Rompin, Johor, Malaysia, the findings revealed that this extract had significant cytotoxic and apoptosis-inducing properties against breast cancer cell lines, including MDA-MB-231 and MCF-7. Notably, fatty acids and other secondary metabolites, including palmitic acid (Compound No. 21, Table 1), which are enriched in the extract, contribute to its antioxidant and antiproliferative activities. However, further investigations are required to elucidate and identify these bioactive compounds, to evaluate their effectiveness in vivo [75].

### 3.5. Alkaloids Compounds

Allantoin (Compound No. 3, Table 1) is the main bioactive component of *Dioscorea* spp. It is primarily synthesized through the alkaloid pathway. The presence of a heterocyclic structure and functional groups within the allantoin structure confer it with both chemical stability and solubility, as well as enabling it to participate in various beneficial biological and physiological functions. Yam-derived allantoin has been shown to possess ameliorative effects by inhibiting apoptosis, autophagy, and pyroptosis in an animal model involving cyclophosphamide-induced premature ovarian failure, thereby emphasizing its potential as a protective agent against premature ovarian failure in clinical practice [131]. In another study, allantoin obtained from the rhizome extract of *Dioscorea batatas* promoted myoblast differentiation and enhanced mitochondrial biogenesis. These effects contributed to a significant increase in glucose uptake and adenosine triphosphate (ATP) production in myotubular cells, indicating that allantoin plays a significant role in the mitigation of age-related sarcopenia and associated diseases [132].

In addition to allantoin, other bioactive alkaloids are synthesized through the same biogenic pathway in *Dioscorea sansibarensis.* Notably, camptothecin (Compound No. 49, Table 1) identified in the aqueous extract of *Dioscorea sansibarensis* Pax, showed cytotoxic effects against HPV-negative and positive head and neck squamous cell carcinoma (HNSCC) cell models. Furthermore, camptothecin enhanced the sensitivity of these cells to radiation therapy, especially in a three-dimensional cell culture model [98]. *Dioscorea bulbifera* extracts exhibited significant bioactive effects, including antimalarial, antiviral, anti-diabetic, and anticancer effects. These effects are believed to arise from the activities of bioactive compounds, including dioscorine (Compound No. 51, Table 1), enhancing its clinical application [100]. Additionally, yramine (Compound No. 36, Table 1), a bioactive ingredient in the aqueous extract of yam (CYW), has been revealed to play a protective role against ethanol-induced gastric injury [63].

### 3.6. Amino Acids and Peptide Compounds

*Dioscoreae* spp. are rich in essential amino acids, including L-tryptophan and L-glutamic acid, which contribute significantly to their high nutritional value [111]. Additionally, some of the amino acids exhibit physiologically active effects. For instance, cycloleucines (Compound No. 37, Table 1) are amino acid compounds obtained from *Dioscoreae Rhizoma* extract and play important physiological roles, including the mitigation of ethanol-induced gastric injury via anti-inflammatory, antioxidant and cytoprotective pathways [63].

## 4. Functional Properties of *Dioscorea* spp.

**Figure 3:**
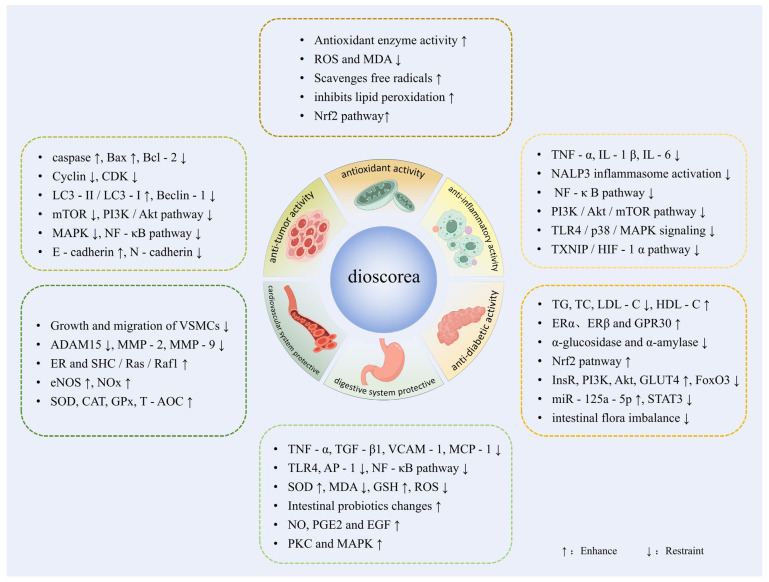
Bioactivities and health benefits associated with *Dioscorea* spp.

The bioactive constituents in *Dioscorea* spp. confer substantial potential to serve as functional foods. Research has widely established that the health properties of plant-based functional foods are closely associated with the functional properties of their plant ingredients. Recently, extensive research has been conducted on the functional properties of *Dioscorea* spp., providing a robust framework for their application as effective functional food ingredients. Figure 3 presents the potential health benefits of *Dioscorea* spp. as a functional food ingredient.

### 4.1. Antioxidant Activity

Epidemiologic studies have shown that regular dietary intake of vegetables and fruits with antioxidant activity offers protective effects to the body against free radicals and reactive oxygen species (ROS), effectively suppressing the progression of chronic diseases, thereby reducing the mortality rate of age-related diseases such as coronary heart disease. Consequently, there has been progressive exploration of alternative therapies that use natural and safe sources of food antioxidants to replace the use of synthetic antioxidants that may exhibit cytotoxicity and potential adverse effects [133]. The majority of studies investigating the functional food potential of *Dioscorea* spp. have predominantly focused on the extraction and compositional analysis of the edible tuberous parts, with comparatively limited attention given to the phytochemical profiling of the above-ground organs. For example, Boudjada et al. evaluated the antioxidant properties of fresh rhizomes of *Dioscorea communis* L. using multiple in vitro assays, including 1,1-diphenyl-2-picrylhydrazyl (DPPH) radical scavenging, 2,2′-azinobis-(3-ethylbenzthiazoline-6-sulphonate) (ABTS) cationic radical decolorization, the CUPric reducing antioxidant capacity (CUPRAC) assay, the reducing power assay, and β-carotene bleaching methods [116]. Flavonoids are polyphenols with antioxidant properties capable of affecting plant color. J. Zhang et al. utilized a novel ionic liquid-based ultrasound-assisted extraction (IL-AEA) technique to extract functional polyphenolic compounds such as flavonoids from purple yam. Among these flavonoids, anthocyanins—natural water-soluble pigments—have gained significant attention due to their strong antioxidant activity [134]. In another study, Qiu et al. used purified anthocyanins obtained from *Dioscorea alata* L. to evaluate their antioxidant activity using the DPPH, ABTS, and Fe^3+^ reducing power assays [135]; the results indicated that the purified anthocyanins had stronger antioxidant activity compared to the unpurified extract and other ascorbic acid.

The leaves of *Dioscorea* spp., which are usually discarded or incinerated as waste, contain an abundant proportion of bioactive secondary metabolites. These metabolites exhibit stronger antioxidant activity [69,79], highlighting the potential of yam leaves as a sustainable source of bioactive compounds. Similarly, the significant quantity of yam peel (specifically Chinese yam peel) is valuable material containing secondary bioactive components; however, these peels are often discarded during food processing as waste. Shao et al. used response surface methodology (RSM) to optimize the extraction conditions and purity levels of polysaccharide (CYPP-1) from the yam peel [136]. This polysaccharide exhibits superior antioxidant activity. Liu et al. investigated the antioxidant activity of the pericarp polysaccharide (DTP) obtained from *Dioscorea oppositifolia* L [137]. They prepared their two metal chelates: iron chelate (DTP-Fe) and zinc chelate (DTP-Zn). By using DPPH, ABTS^+^, and hydroxyl radical scavenging assays, they demonstrated that both DTP and its chelates possessed strong antioxidant activities, with DTP-Fe exhibiting the strongest antioxidant capacity. Other bioactive components contained in yam skin may contribute to its antioxidant activity. Repurposing yam leaves and peels, which are often regarded as agricultural byproducts, provides therapeutic solutions while also addressing environmental concerns; specifically, these products allow for the production of antioxidant-rich products. Consequently, they offer substantial progress in the industrial production of functional foods.

Zhou et al. purified yam polysaccharides (PYB) from the bead buds of *Dioscorea opposita* Thunb. They obtained two active β-configurations, PYB-1 and PYB-2, with approximate molecular weights of 145 and 11 k Da, respectively [44]. These bioactive compounds may serve as potential natural antioxidants in the functional food industry [138]. Additionally, other studies have focused on the antioxidant activity of crude polysaccharides as opposed to purified polysaccharides [139,140].

The antioxidant properties of *Dioscorea* spp. are closely related to a variety of bioactive components, such as diosgenin elements, structurally characterized proteins, and bioactive peptides. Research has shown that diosgenin elements exhibit moderate antioxidant activities in vitro [23]. Jegadheeshwari et al. extracted and purified a trypsin inhibitor (Db GTi protein) from *Dioscorea bulbifera* L. tubers. Subsequently, they used several antioxidant assays on Db GTi protein and their results demonstrated excellent antioxidant activity, with the ability to protect against Cr (VI)-induced oxidative stress [141,142]. Additionally, they demonstrated that it was non-toxic and inhibited the activity of pathogenic microorganisms such as Klebsiella pneumoniae. Lectins, a class of proteins or glycoproteins, exhibit a diverse array of potential biological activities, including antioxidant, and have therefore gained significant attention in recent years. Studies have shown that *Dioscorea tuberosa* hemagglutinin exhibits potent antioxidant properties and could serve as a potential resource for the development of functional or health foods, as well as a significant target in food protein studies [143,144].

### 4.2. Anti-Inflammatory and Immunomodulatory Activity

Gouty arthritis (GA) is an inflammatory joint disease caused by the deposition of monosodium urate (MSU). Notably, GA significantly impacts the quality of life and daily work efficiency of patients. Traditional Western drug treatments, such as colchicine, can relieve symptoms; however, these treatments have been associated with significant adverse events. In recent years, traditional Chinese medicine (TCM) has been the preferred treatment option for most patients due to its superior efficacy and fewer side effects [145]. Extensive experiments conducted by Zhou et al. showed that *Dioscorea nipponica* Makino total saponin exhibits potential in treating GA by regulating multiple signaling pathways, such as lysosomal enzymes, antioxidant capacity, and NALP3 inflammatory vesicles, and regulating arachidonic acid [146,147,148,149,150]. Additional studies have demonstrated that total saponins extracted from *Dioscorea* spp. can attenuate MSU crystal-induced inflammation by inhibiting the activation of NLRP3 inflammasomes and caspase-1, both of which are stimulated by fisetinone. These findings suggest that NLRP3 and caspase-1 may serve as novel therapeutic targets for the treatment of GA [151]. Several studies have shown that the saponin fractions of *Dioscorea* spp. also exert anti-hyperuricemia activity through blood uric acid-lowering effects, thereby preventing and reducing GA attacks [105,152,153].

In neurodegenerative diseases, activation of microglia due to endogenous or exogenous injury leads to neuroinflammation. Research has established that diosgenin from *Dioscorea nipponica* Makino could protect BV-2 microglia from LPS-activated inflammatory responses by inhibiting NF-κB phosphorylation and up-regulating brain-derived neurotrophic factors in the cerebral cortex and hippocampal regions of the mouse brain, consequently ameliorating the progression of several neurodegenerative diseases [29]. Through the isolation and characterization of relevant compounds, phenanthrene derivatives identified in *Dioscorea batatas* and *Dioscorea bulbifera* have been found to exert anti-inflammatory effects by inhibiting LPS-mediated inflammatory responses in BV2 cells [154,155]. Anti-inflammatory effects in *Dioscorea* spp. provide novel ideas for the treatment of inflammation-related neurological disorders and the development of associated functional foods for the elderly.

In recent years, the immunomodulatory properties of *Dioscorea* spp. have been extensively investigated to facilitate the development of functional foods. Polysaccharide fractions extracted from *Dioscorea opposita* Thunb and *Dioscorea batatas* Decne exhibit significant potential benefits in enhancing immune responses [42,130,136,156,157]. Consequently, the polysaccharides and glycoproteins in *Dioscorea* spp. exhibit significant potential as immune enhancers in the development of functional foods.

### 4.3. Anti-Diabetic and Endocrine Modulating Activity

**Figure 4:**
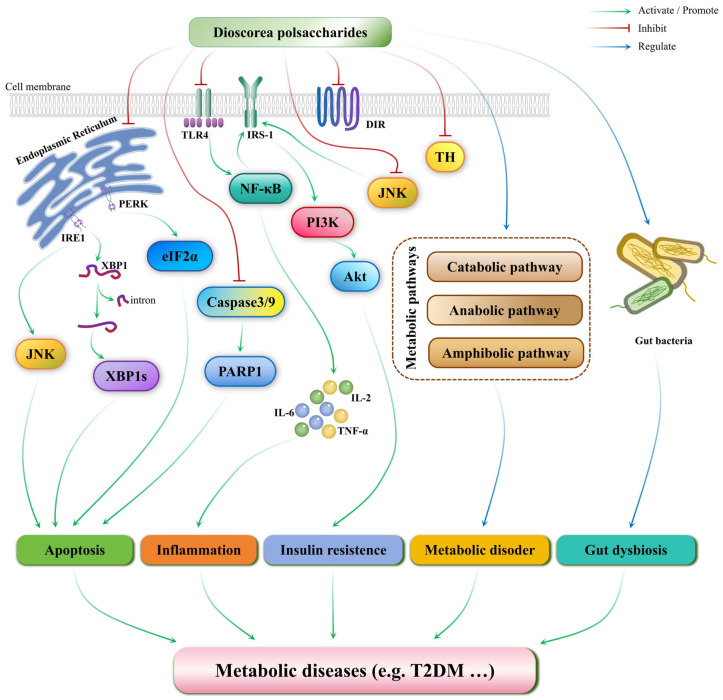
The functional pathways of *Dioscorea* polysaccharides in metabolic disorders.

Feng et al. isolated and characterized an acidic polysaccharide (CYPB) from Chinese yam [158]. The experimental results involving a high-fat diet and streptozotocin-induced Type 2 Diabetes Mellitus (T2DM) mouse model indicated that CYPB may improve T2DM and alleviate the symptoms of impaired glucose tolerance. These effects were due to its ability to regulate the PI3K/Akt signaling pathway, increasing glycogen synthesis, reducing gluconeogenesis, and improving insulin resistance. Additionally, Fan et al. established tetracosan-induced diabetic rat and mouse models to evaluate the in vivo hypoglycemic activity of *Dioscorea opposita* Thunb. polysaccharides (DOTPs) [159]. Figure 4 presents the underlying mechanism of action of *Dioscorea* polysaccharides in ameliorating metabolic diseases such as T2DM. Other bioactive constituents that substantially contribute to the anti-diabetic activity of *Dioscorea* spp. include steroidal saponins, allantoin, phenanthrenes, and diarylheptanoids. Steroidal saponins, such as dioscin, have been shown to improve both fasting and postprandial hyperlipidemia and demonstrate significant anti-diabetic activity against type 2 diabetes mellitus (T2DM). These effects are mediated, in part, through the regulation of glucolipid metabolic disorders and the inhibition of α-glucosidase activity, among other mechanisms [28,33,77,160]. Analysis of different yam starches found that *Dioscorea opposita* Thunb. starch exhibited the lowest glycemic index. Additionally, resistant starch derived from *Dioscorea alata* L. improved lipid metabolism by modulating intestinal flora. These results indicate that yam starch is an excellent source of starch for functional foods and is particularly suitable for dietary use in diabetic populations [85,86]. Matsuokad et al. conducted an open-label, randomized crossover trial involving 14 healthy Japanese adults using yam paste. The results showed that consuming barley mixed with rice and yam paste significantly reduced postprandial blood glucose concentrations and insulin secretion, providing clinical evidence for the development of Dioscorea-based functional foods for blood sugar control [161].

Additionally, *Dioscorea opposita* Thunb. protects the male reproductive system through endocrine regulation. Specifically, in a rat model of hydrocortisone-induced erectile dysfunction (ED), *Dioscorea opposita* Thunb. cold infusion extract (CYCSE) stimulated testosterone secretion and increased the proliferation activity of Leydig cells, thereby protecting testicular morphology, restoring erectile function, and exerting therapeutic effects on ED [162]. These findings hold significant value for the advancement of functional foods aimed at addressing male infertility caused by diabetes, given the rising incidence of male infertility. Additionally, *Dioscorea* spp. potentially regulates hormones, resulting in better therapeutic outcomes in metabolic disorders among women. The first protein-based therapeutic agent for menopausal syndrome was a protein termed DOI, which was isolated from *Dioscorea opposita* Thunb. It induces estradiol and progesterone secretion by up-regulating the expression of follicle-stimulating hormone receptors and ovarian aromatase. These effects have also been shown not to result in either breast or ovarian cancer, rendering it a safe and highly effective alternative approach to hormone replacement therapy (HRT) [163,164]. Due to its estrogen-like effects and ability to offer ovarian protection [131,165], *Dioscorea opposita* Thunb. exhibits a positive impact on the health of women, especially in estrogen level-related health problems such as menopausal symptoms and premature ovarian failure. Related functional foods for women based on *Dioscorea opposita* Thunb. are important for the protection of female reproductive health, treatment of female aging disorders, and prevention of related diseases.

### 4.4. Digestive System Protective Activity

With the increasing prevalence of digestive disorders associated with modern fast-paced lifestyles, the potential of *Dioscorea* species in addressing inflammatory conditions of the gastrointestinal tract—especially hepatitis, pancreatitis, and inflammatory bowel disease (IBD)—is gradually becoming well-characterized through emerging research. Koo et al. explored the anti-inflammatory effects of *Dioscorea batatas* Decne extract on the livers of Western diet-fed ApoE(−/−) mice and LPS-activated HepG2 cells [166]. The results suggest that *Dioscorea batatas* Decne extract attenuates hepatic inflammation and fibrosis by inhibiting the TLR4-AP1-mediated signaling pathway. Multiple studies have reported that the saponin constituents of *Dioscorea* spp. have hepatoprotective effects [36,167]. Therefore, *Dioscorea* spp. has good potential for application as a functional food plant source with hepatoprotective effects in patients with chronic liver inflammation. A literature review on acute pancreatitis (AP) revealed that most studies have focused on a single species of *Dioscorea zingiberensis* [65,168,169]. In the gastric environment, several constituents of the aqueous extract of Chinese yam (*Dioscorea opposita*)—including yam polysaccharides, linoleic acid, 3-acetyl-11-keto-β-lactobionic acid, and adenosine—have been shown to prevent and alleviate ethanol-induced gastric injury through anti-inflammatory, antioxidant, and anti-apoptotic mechanisms [47,63]. This indicates that Chinese yam may serve as a natural source for the development of functional foods with the potential to improve gastric health.

Dioscin, a natural active product of *Dioscorea nipponica* Makino, inhibits AOM/DSS-induced colitis, thereby preventing the progression of colon cancer [170]. Network pharmacological analyses have shown that *Dioscorea nipponica* Makino can potentially treat IBD through the Ras-MAPK signaling and the PI3K-Akt signaling pathways [171]. Previous research has demonstrated that polyphenols represented by anthocyanins in yam exert potent anti-inflammatory effects through the inhibition of the NF-κ B and STAT3 signaling pathways, thereby preventing and alleviating ulcerative colitis and the associated colorectal cancer [73,122,172]. In addition to the anti-inflammatory effects of polyphenols, a subset of glycoproteins and phenanthrenes in yam can offer protection from damage to the intestinal mucosa and prevent IBD by promoting cell migration signaling events, as well as influencing the production of inflammatory cytokines through the NF-κB pathway [156,173]. In addition to anti-inflammatory effects through signaling pathways, optimal IBD treatment demands the modulation of intestinal flora and maintenance of intestinal homeostasis. Building on this foundation, Mu et al. investigated the modulatory effects of anthocyanins derived from purple yam on gut microbiota and elucidated their potential role in the pathogenesis and mitigation of IBD [174]. These findings provide evidence-based support for the use of *Dioscorea* as a natural functional food to ameliorate IBD and regulate intestinal health.

### 4.5. Cardiovascular System Protective Activity

*Dioscorea opposita* Thunb., along with its constituent adenosine, exhibits estrogen-like effects and offers protective effects against LPS-induced myocardial dysfunction. Specifically, *Dioscorea opposita* Thunb. extract and adenosine attenuated LPS-induced myocardial dysfunction by inhibiting the renin–angiotensin system and apoptosis through activation of the estrogen receptor-mediated SHC/Ras/Raf1 signaling pathway [46]. These findings support the potential application of functional foods containing natural estrogens from *Dioscoreae* to mitigate cardiovascular diseases, especially those due to menopause or sepsis in women. *Dioscoreae Nipponicae* Rhizoma is an established medicinal herb used in the treatment of myocardial ischemia (MI). It exhibits significant potential for the development of pharmaceutical agents and application in the functional food market. Diosgenin has been identified as a likely key bioactive constituent of *Dioscoreae Nipponicae Rhizoma*, responsible for its cardioprotective effects against MI [25]. Yang et al. conducted an analytical and comparative study involving *Dioscoreae Nipponicae* Rhizoma from varying geographic origins using UPLC-Q time-of-flight metabolomics coupled with molecular docking technology, further confirming that this compound has the potential to be used in the treatment of MI, irrespective of origin [175]. *Dioscorea panthaica* Prain et Burkill and *Dioscorea zingiberensis* C.H. Wright have demonstrated anti-MI activities comparable to those observed in *Dioscoreae Nipponicae* Rhizoma. [176]. This finding indicates that all three *Dioscoreae* could be used as sources of drugs for MI and the development of functional foods.

Additionally, *Dioscorea* can ameliorate symptoms and disorders not typically classified as cardiovascular diseases, but that are closely related to them, such as aplastic anemia [177] and restenosis (or neoplastic endothelial hyperplasia) [66]. Cerebral ischemia/reperfusion (I/R) injury is a secondary complication in ischemic stroke patients who have undergone thrombolysis or revascularization. Currently, an increasing number of natural plant constituents are being identified as exhibiting positive effects on the treatment of I/R injury, such as notoginsenoside R1 [178]. Additionally, steroidal saponin constituents from *Dioscorea* spp. have been shown to have potential I/R protective effects. Steroidal saponins have been shown to mitigate ischemia/reperfusion (I/R)-induced excessive autophagy and inflammatory responses by modulating the PI3K/AKT/mTOR signaling pathway. These compounds help preserve the integrity of the blood–brain barrier (BBB) and exert neuroprotective effects, thereby significantly improving neurological outcomes and reducing brain injury in I/R models [60,179,180,181]. Diosgenin exhibits potential for development as a plant-derived ingredient in dietary supplements or functional foods, offering a promising natural approach for preventing and mitigating I/R.

### 4.6. Anti-Tumor Activity

*Dioscorea* species have demonstrated potential for use in the development of functional foods, particularly in response to female-relevant tumors such as breast cancer. The cytotoxic effects of *Dioscorea bulbifera* were evaluated using the 3-(4, 5-dimethylthiazol-2-yl)-2, tetramethyl azole salts (MTT) assay, assessing its impact on MDA-MB-231 and MCF-7 breast cancer cell lines. The extract was found to significantly promote apoptosis in both cell lines [75]. This result suggests that *Dioscorea bulbifera* extract can be used as a promising natural agent for combating breast cancer invasiveness. Additionally, the steroidal saponins in *Dioscorea bulbifera*, such as dioscin, have potent anti-breast cancer effects [182]. Structural modifications of the *Dioscorea bulbifera* molecular framework, including the opening of the spirocyclic ketone bond and the synthesis of carbamate derivatives at the C-26 position of the furostene ring, have led to the creation of novel compounds [26]. For instance, various isoxazole derivatives have been synthesized by modifying the hydroxyl group at the C-3 position [183]. The related analogs from these modifications have shown significant antioxidant and antiproliferative effects against human breast cancer cell lines, coupled with favorable safety and tolerability profiles, indicating their potential for optimization as more effective anti-breast cancer compounds. Beyond breast cancer, *Dioscorea* spp. have shown notable tumor-inhibitory effects against ovarian and cervical cancers [45,98,184].

### 4.7. Other Functional Activities

Moreover, *Dioscorea opposita* Thunb. polysaccharide has exhibited anti-fatigue effects, especially cancer-caused fatigue. In forceful swimming experiments in mice, it was observed that the polysaccharide significantly prolonged the swimming time and exhibited effective anti-fatigue properties [44,185]. This provided evidence that yam polysaccharides may serve as functional food additives to relieve fatigue. *Dioscorea* spp. can improve memory and cognitive function, as well as enhance the resolution of depression and Alzheimer’s disease [71]. Tohdad et al. conducted a randomized, double-blind, placebo-controlled, crossover trial (28 healthy adults aged 20–81 years) and demonstrated for the first time that yam extract rich in diosgenin can safely and effectively enhance cognitive function in healthy adults (RBANS total score +4.25 points, *p* = 0.0129), with more pronounced effects in individuals aged 47 years and older, offering a new strategy for AD prevention or early intervention [186]. This is largely attributable to the pharmacological effects of diosgenin [187]. The use of functional foods containing *Dioscorea batatas* in the elderly can restore the loss of skeletal muscle mass and dysfunction associated with aging [132]. Keikod et al. conducted a 12-week double-blind randomized controlled trial to evaluate the effects of *Dioscorea esculenta* on 60 middle-aged and elderly individuals (53 ± 5 years old). The study confirmed that *Dioscorea* combined with low-intensity training can improve muscle mass and quality and metabolic health in middle-aged and elderly individuals [188]. Researchers have also shown that *Dioscorea batatas* can promote the repair of skeletal defects, with potential to exert osteoprotective effects in a rat model of alveolar bone loss caused by ovariectomy [189]. Moreover, the osteoprotective effect of *Dioscorea* may be mediated by its osteogenic proteins [190]. We believe that the osteogenic proteins in *Dioscorea* spp. could serve as osteoinducers in functional foods to treat and protect against bone defects.

## 5. Functional Food Regulation

Functional foods are a category of foods that contain specific functional ingredients and, in addition to providing basic nutrition, may have potential positive effects on health [1]. Functional foods are increasingly becoming a hot topic in food development and consumption, offering users an ever-growing range of positive effects. Regardless of the region, health claims for functional foods must be supported by scientific evidence, experimental data, human clinical trials, and safety assessments [191]. However, each country or region has its own regulatory frameworks and policies for functional foods, such as the Food and Drug Administration (FDA) and the European Food Safety Authority (EFSA). China has established a food safety management system centered on the “Food Safety Law of the People’s Republic of China” to regulate functional foods. Countries like the United States have enacted specific legislation to regulate functional foods. The European Union has implemented Directive 2002/46/EC of the European Parliament, promoting cross-border assessments through data-sharing mechanisms [192].

In China, yam from the *Dioscorea* genus is currently permitted as a functional food ingredient, but it must comply with the requirements of the “Food and Medicine Homology Directory” and complete registration or filing in accordance with functional claims, providing safety and efficacy trial evidence. For other *Dioscorea* species, approval as a new food ingredient is required, and they may not be used in food without approval [193,194]. In the United States, *Dioscorea* and its extracts are regulated as conventional food or dietary supplements. Fresh or dried products are considered food, while extracts must comply with the Dietary Supplement Health and Education Act (DSHEA), allowing claims of “supporting health” but not treatment, and labels must include a disclaimer. Traditional ingredients are considered Generally Recognized as Safe (GRAS), while new ingredients must be reported to the FDA 75 days in advance [195,196]. The EU currently classifies functional foods derived from *Dioscorea* species under either “novel foods” or “traditional herbal medicines”. If there is no significant history of consumption prior to 1997, they must undergo a two-tier safety assessment by EFSA, including toxicological data. Health claims must be clinically validated by EFSA. Notably, varieties containing toxic saponins may be banned, and approved products are subject to daily intake limits [197]. There are significant differences in management and assessment methods for functional foods worldwide, but establishing a standardized international framework could enhance their effectiveness and global safety.

## 6. Conclusions and Perspectives

*Dioscorea* spp., a functional food source with great market potential, contains a wide range of bioactive components, including diosgenin elements. It exerts diverse effects, such as antioxidant and anti-inflammatory activity, endocrine modulation, and neuroprotection. To date, several investigations have been conducted on *Dioscorea* spp. and their bioactive components. However, given the diversity of *Dioscorea* species and the numerous bioactive components they contain, there are gray areas that have not been fully explored. Therefore, future research on *Dioscorea* foods should focus on specific species within the genus, including their unique potential functional characteristics or notable toxicity. For example, certain species of *Dioscorea* contain toxic components that may induce liver toxicity. Before these species can be developed into functional foods, relevant toxicological studies must be conducted to ensure food safety. In recent years, attempts have been made to improve the physiological effects of active ingredients in *Dioscorea* spp. This has led to a surge in research focusing on novel techniques to improve the functionality of *Dioscorea* spp. foods, such as the utilization of exosomes and other innovative methods.

*Dioscorea* spp. are well known for their antioxidant, anti-inflammatory, and preventive potential against several diseases. The abundant polysaccharides, saponins, polyphenols and vitamins contribute to the maintenance of human health and well-being, especially in the elderly and women’s health. If *Dioscorea* functional foods are to be fully exploited, they should be explored continuously. It is essential not only to isolate and identify the structure of the bioactive compounds but also to conduct clinical trials to confirm their therapeutic effects. This will help to determine the recommended serving sizes and durations of effect of *Dioscorea* functional foods. In the future, the functional food industry for the Dioscorea genus will need to fully consider technical, sensory, and food safety aspects to develop the optimal methods for incorporating Dioscorea or related components into foods. Additionally, the characteristic properties of such products will require further exploration and validation through extensive clinical trials to confirm their health benefits and efficacy for human consumption.

## Figures and Tables

**Table 1:** Phytochemicals present in *Dioscorea* spp. cand their structural classification and functional activity.

Sl No.	Phytoconstituents	Nature	Species	Functional Activity	Ref.
1	Diosgenin	Terpenoids—Steroids	*Dioscorea bulbifera*, *Dioscorea nipponica*, *Dioscorea zingiberensis*, *Dioscorea esculenta*, *Dioscorea cirrhosa* L., *Dioscorea japonica*, *Dioscorea opposita* Thunb.	Antioxidant, anti-inflammatory, anti-tumor, antibacterial, anti-hyperlipidemia, anti-myocardial ischemia, regulation of intestinal flora, hypoglycemia, neuroprotection, treatment of thyroid disease, etc.	[19,20,21,22,23,24,25,26,27,28,29]
2	Dioscin	Terpenoids—Steroids	*Dioscorea nipponica* Makino, *Dioscorea spongiosa*, *Dioscorea alata* L., *Dioscorea opposita* Thunb., *Dioscorea bulbifera*, *Dioscorea japonica*	Hypoglycemic, anti-tumor, anti-inflammatory, neuroprotective, hepatoprotective, treatment of thyroid disease, etc.	[30,31,32,33,34,35]
3	Allantoin	Alkaloids—Pseudoalkaloids	*Dioscorea belophylla*, *Dioscorea batatas*, *Dioscorea opposita* Thunb, *Dioscorea deltoidea*	Improve pancreatic islet damage, anti-premature ovarian aging, hypoglycemia, antioxidant, anti-tumor, regulation of intestinal flora, prevention of skeletal muscle dysfunction, etc.	[36,37,38,39,40,41]
4	Polysaccharides	Carbohydrates	*Dioscorea opposita* Thunb., *Dioscorea bulbifera*, *Dioscorea nipponica* Makino, *Dioscorea polystachya* Turcz.	Immune regulation, anti-inflammatory, flora regulation, anti-diabetic and obesity, anti-fatigue, antioxidant, cardioprotection, anti-tumor, digestive system protection, etc.	[42,43,44,45,46,47]
5	Gracillin	Terpenoids—Steroids	*Dioscorea spongiosa*, *Dioscorea quinqueloba*, *Dioscorea tokoro* Makino	Anti-allergic, anti-tumor, anti-inflammatory, inhibit melanogenesis, lower uric acid, anti-diabetic	[48,49,50,51]
6	Protodioscin	Terpenoids—Steroids	*Dioscorea deltoidea*, *Dioscorea nipponica* Makino, *Dioscorea tokoro*	Anti-tumor, hepatoprotective, anti-inflammatory, anti-leukemia	[52,53,54]
7	Diosbulbin B	Terpenoids—Diterpenoids	*Dioscorea bulbifera* L.	Induces hepatotoxicity	[55,56]
8	Dioscorin	Alkaloids—Lysine alkaloids	*Dioscorea alata*, *Dioscorea bulbifera* L.	Hypoglycemia, insecticide, etc.	[28,57]
9	Pseudoprotodioscin	Terpenoids—Steroids	*Dioscorea septemloba* Thunb., *Dioscorea spongiosa*, *Dioscorea nipponica* Makino	Anti-tumor, hepatoprotective, anti-inflammatory	[53,58]
10	Deltonin	Terpenoids—Steroids	*Dioscorea zingiberensis*	Therapeutic I/R, anti-tumor, hepatoprotective	[59,60]
11	Myricetin	Shikimates and Phenylpropanoids—Flavonoids	*Dioscorea alata*, *Dioscorea bulbifera*	Anti-HIV-1 integrase activity, anti-inflammatory, pro-wound healing, antioxidant	[19,61,62]
12	Adenosine	Carbohydrates—Nucleosides	*Dioscorea opposita* Thunb., *Dioscorea polystachya*	Cardioprotection, ex vivo and ex vivo estrogenic effects, anti-inflammatory, etc.	[63,64,65]
13	Protogracillin	Terpenoids—Steroids	*Dioscorea nipponica* Makino, *Dioscorea septemloba* Thunb	Anti-tumor, anti-inflammatory, uric acid-lowering, anti-diabetic	[31,53,65]
14	Methyl protodioscin	Terpenoids—Steroids	*Dioscorea nipponica* Makino, *Dioscorea villosa*, *Dioscorea spongiosa*	Anti-tumor, inhibits neoplastic endothelial formation	[66,67]
15	Quercetin	Shikimates and Phenylpropanoids—Flavonoids	*Dioscorea opposite*, *Dioscorea alata*, *Dioscorea bulbifera* L., *Dioscorea glabra* Roxb.	Anti-inflammatory, antimalarial, antioxidant, antiviral	[68,69,70]
16	Yamogenin	Terpenoids—Steroids	*Dioscorea collettii*	Anti-tumor, antioxidant, antibacterial, antidepressant	[23,71]
17	Anthocyanins	Shikimates and Phenylpropanoids—Flavonoids	*Dioscorea alata* L.	Anti-inflammatory, regulates intestinal flora, antioxidant	[72,73]
18	Cholesterol	Terpenoids—Steroids	*Dioscorea bulbifera*, *Dioscorea zingiberensis*	Anti-tumor, antioxidant	[74,75]
19	batatasin I	Shikimates and Phenylpropanoids—Phenanthrenoids	*Dioscorea opposita* Thunb., *Dioscorea dumetorum* (Kunth)	Antioxidant, anti-diabetic	[76,77]
20	Epicatechin	Shikimates and Phenylpropanoids—Flavonoids	*Dioscorea cirrhosa* L.	Antioxidant, etc.	[78,79]
21	Palmitic acid	Fatty acids —Fatty Acids and Conjugates	*Dioscorea japonica* Thunb., *Dioscorea bulbifera*	Acaricidal properties, etc.	[75,80]
22	Rutin	Shikimates and Phenylpropanoids—Flavonoids	*Dioscorea ploystchya* Turcz., *Dioscorea bulbifera* L.	Antioxidant, etc.	[69,79,81]
23	Luteolin	Shikimates and Phenylpropanoids—Flavonoids	*Dioscorea ploystchya* Turcz., *Dioscorea bulbifera* L.	Antioxidant, antiviral	[70,79]
24	Dioscorealide B	Shikimates and Phenylpropanoids—Phenanthrenoids	*Dioscorea membranacea* Pierre	Anti-tumor, etc.	[82,83]
25	Resistant Starch	Carbohydrates	*Dioscorea alata L.*, *Dioscorea opposita* Thunb.	Regulate intestinal flora, anti-hyperlipidemia, anti-hyperglycemia and obesity	[84,85,86]
26	gamma-aminobutyric acid	Amino acids and Peptides —Small peptides	*Dioscorea polystachya* Turczaninow	Antihypertensive, anti-anxiety, anti-diabetic, etc.	[87,88]
27	Glutamine	Amino acids and Peptides —Small peptides	*Dioscorea polystachya*	Antioxidant, etc.	[64,89]
28	Paeonol	Shikimates and Phenylpropanoids	*Dioscorea japonica* Thunb.	Anti-inflammatory, neuroprotective, antioxidant, anti-tumor, etc.	[90,91]
29	Stigmasterol	Terpenoids—Steroids	*Dioscorea alata*	Antioxidant, anti-hyperlipidemia	[19,92]
30	Huangjiangsu A	Terpenoids—Steroids	*Dioscorea zingiberensis*, *Dioscorea villosa*	Antioxidant, liver protection	[36,93]
31	Taxifolin	Shikimates and Phenylpropanoids—Flavonoids	*Dioscorea opposita* Thunb.	Regulates intestinal flora and synergistically enhances short-chain fatty acid production	[27]
32	Protodeltonin	Terpenoids—Steroids	*Dioscorea villosa*	Antioxidant, liver protection	[36]
33	Tokoronin	Terpenoids—Steroids	*Dioscorea tokoro* Makino	Low cytotoxicity, inhibits melanogenesis	[51]
34	kaempferol	Shikimates and Phenylpropanoids—Flavonoids	*Dioscorea ploystchya* Turcz., *Dioscorea bulbifera* L.	Anti-inflammatory, antioxidant, anti-lipogenic	[62]
35	Lactulose	Carbohydrates—Saccharides	*Dioscorea rhizoma*	Synergistic anti-ethanol gastric injury	[63]
36	Tyramine	Alkaloids—Tyrosine alkaloids	*Dioscorea polystachya*	Synergistic anti-ethanol gastric injury	
37	Cycloleucine	Amino acids and Peptides —Small peptides	*Dioscorea polystachya*	Synergistic anti-ethanol gastric injury	
38	Rosmarinic acid	Shikimates and Phenylpropanoids—Phenolic acids (C6-C1)	*Dioscorea glabra* Roxb., *Dioscorea alata*	Antioxidant, etc.	[69]
39	Phytol	Terpenoids—Diterpenoids	*Dioscorea bulbifera*	Anticancer, antioxidant	[75]
40	muristerone A	Terpenoids—Steroids	*Dioscorea dumetorum* (Kunth) Pax.	Anti-diabetic, strong α-amylase inhibitory activity	[77]
41	dihydroresveratrol	Shikimates and Phenylpropanoids—Stilbenoids	*Dioscorea dumetorum* (Kunth) Pax.	Anti-diabetic, strong α-glucosidase inhibitory activity	
42	Kaempferide	Shikimates and Phenylpropanoids—Flavonoids	*Dioscorea ploystchya* Turcz., *Dioscorea bulbifera* L.	Antioxidant, etc.	[79]
43	Octanoic acid	Fatty acids —Fatty Acids and Conjugates	*Dioscorea japonica* Thunb.	Acaricidal properties, etc.	[80]
44	Beta-sitosterol	Terpenoids—Steroids	*Diascorea alata*	Synergistic relief of gout and its complications, anti-inflammatory, antioxidant, anti-hyperlipidemia, relief of female menopausal symptoms	[92]
45	Peonidin	Shikimates and Phenylpropanoids—Flavonoids	*Dioscorea alata* L.	Powerful antioxidant activity	[94]
46	Cyanidin	Shikimates and Phenylpropanoids—Flavonoids	*Dioscorea alata* L.	Powerful antioxidant activity	[95]
47	Spiroconazol A	Terpenoids—Steroids	*Dioscorea bulbifera* L.	Treatment of non-small cell lung cancer	[96]
48	montroumarin	Shikimates and Phenylpropanoids—Coumarins	*Dioscorea collettii*, *Dioscorea septemloba* Thunb.	Synergistic relief of gout and its complications, anti-inflammatory, analgesic	[97]
49	Camptothecin	Alkaloids—Tryptophan alkaloids	*Dioscorea sansibarensis* Pax.	Anti-head and neck squamous cell carcinoma	[98]
50	Flavanthrinin	Shikimates and Phenylpropanoids—Phenanthrenoids	*Dioscorea bulbifera* L.	Treatment of skin infections, powerful antimicrobial action and low cytotoxicity	[99]
51	Dioscorine	Alkaloids—Lysine alkaloids	*Dioscorea bulbifera*	Blockade of nicotinic acetylcholine receptor, antibacterial and insecticidal activity	[100]
52	Diosbulbin C	Terpenoids—Diterpenoids	*Dioscorea bulbifera* L.	Treatment of non-small cell lung cancer	[101]
53	Prosapogenin A	Terpenoids—Steroids	*Dioscorea zingiberensis*	Stronger anti-tumor activity, hepatoprotective, low hemolytic effect	[102]
54	Diospongin B	Shikimates and Phenylpropanoids—Diarylheptanoids	*Dioscorea spongiosa*	Anti-inflammatory, anti-leishmanial, anti-fungal	[103]
55	Preussinate	Shikimates and Phenylpropanoids—Flavonoids	*Dioscorea preussii* Pax.	Antioxidant, urease inhibitory properties	[104]
56	Tigogenin	Terpenoids—Steroids	*Dioscorea spongiosa*	Antihyperuricemic activity in vivo	[105]
57	2,5,6-Trihydroxy-3,4-dimethoxy-9,10-dihydrophenanthrene	Shikimates and Phenylpropanoids—Phenanthrenoids	*Dioscorea bulbifera* L.	DPPH free radical scavenging activity comparable to vitamin C	[106]
58	3,5-dimethoxy-2,7-phenanthrenediol	Shikimates and Phenylpropanoids—Phenanthrenoids	*Dioscorea oppositifolia*	Anti-obesity, inhibits eating efficiency and fat absorption	[107]
59	Methyl Stearate	Fatty acids—Fatty esters	*Dioscorea alata*, *Dioscorea batatas*	Anticancer, etc.	[108]
60	Lidocaine	Alkaloids	*Dioscorea depauaperata*, *Dioscorea glabra*	Anticancer, insecticide	[109]

Notes: Compounds No. 1–30 were the more frequently occurring bioactive compounds derived from literature frequency analysis, and these compounds were mentioned in at least two independent studies and have significant bioactivity. Compounds No. 31–60 have a relatively low frequency of occurrence in the literature, but were included after comprehensive evaluation due to their unique structures or novel mechanisms of bioactive action, with a view to providing a more comprehensive view of *Dioscorea* spp. in functional foods and other potential applications.

## Data Availability

No new data were created or analyzed in this study. Data sharing is not applicable to this article.
